# Ultrasensitive Magnetic Nanoparticle Detector for Biosensor Applications

**DOI:** 10.3390/s17061296

**Published:** 2017-06-06

**Authors:** Yu-Chi Liang, Long Chang, Wenlan Qiu, Arati G. Kolhatkar, Binh Vu, Katerina Kourentzi, T. Randall Lee, Youli Zu, Richard Willson, Dmitri Litvinov

**Affiliations:** 1 Department of Chemical & Biomolecular Engineering, University of Houston, Houston, TX 77204, USA; yliang7@uh.edu (Y.-C.L.); bvvu@uh.edu (B.V.); edkourentzi@uh.edu (K.K.); willson@uh.edu (R.W.); 2 Center for Integrated Bio & Nano Systems, University of Houston, Houston, TX 77204, USA; lvchang@Central.UH.EDU (L.C.); wqiu@uh.edu (W.Q.); 3 Department of Electrical & Computer Engineering, University of Houston, Houston, TX 77204, USA; 4 Materials Science & Engineering, University of Houston, Houston, TX 77204, USA; 5 Department of Chemistry, University of Houston, Houston, TX 77204, USA; akolhatkar@uh.edu (A.G.K.); trlee@uh.edu (T.R.L.); 6 Department of Pathology and Genomic Medicine, Houston Methodist Hospital, Houston, TX 77030, USA; yzu@houstonmethodist.org; 7 Centro de Biotecnología FEMSA, Departamento de Biotecnología e Ingeniería de Alimentos, Tecnológico de Monterrey, Monterrey, NL 64849, Mexico

**Keywords:** bioinstrumentation, biosensor, magnetic particle detection, magnetoresistive sensors

## Abstract

Ta/Ru/Co/Ru/Co/Cu/Co/Ni_80_Fe_20_/Ta spin-valve giant magnetoresistive (GMR) multilayers were deposited using UHV magnetron sputtering and optimized to achieve a 13% GMR ratio before patterning. The GMR multilayer was patterned into 12 sensor arrays using a combination of e-beam and optical lithographies. Arrays were constructed with 400 nm × 400 nm and 400 nm × 200 nm sensors for the detection of reporter nanoparticles. Nanoparticle detection was based on measuring the shift in high-to-low resistance switching field of the GMR sensors in the presence of magnetic particle(s). Due to shape anisotropy and the corresponding demag field, the resistance state switching fields were significantly larger and the switching field distribution significantly broader in the 400 nm × 200 nm sensors as compared to the 400 nm × 400 nm sensors. Thus, sensor arrays with 400 nm × 400 nm dimensions were used for the demonstration of particle detection. Detection of a single 225 nm Fe_3_O_4_ magnetic nanoparticle and a small number (~10) of 100 nm nanoparticles was demonstrated. With appropriate functionalization for biomolecular recognition, submicron GMR sensor arrays can serve as the basis of ultrasensitive chemical and biological sensors.

## 1. Introduction

Giant magnetoresistance (GMR) [1,2] based devices have been studied extensively and are used today in every hard disk drive [3,4,5] as well as in emerging applications such as magnetic random access memory (MRAM) [6,7,8]. One of the newer applications of GMR technology is in biosensors used for detection of biological and biomolecular agents such as virus particles, proteins, bacteria, or nucleic acids [9,10,11,12,13]. Significant progress has been achieved in magnetic biosensor technology since the concept was first proposed by Baselt et al. [9] using micrometer scale GMR sensors through improved control over sensor materials to control domain wall noise and utilization of complex measurements and signal processing methods [12,13,14,15,16,17,18,19,20,21,22,23]. The relatively large sensor-size-to-nanoparticle-size ratios (historically, the ratio of GMR sensor area to a particle area is far in excess of 100) makes sensing of individual nanoparticles or even sensing of a small number of nanoparticles challenging. For example, (1.5–10 μm) × (200–750 nm) sensors used to demonstrate sensing of clusters (hundreds down to several dozens) of 16–50 nm Fe_3_O_4_ nanoparticles utilize Wheatstone bridge to enable ultra-high resolution resistance measurements (0.001% or 10 PPM) [11,12,16,18,24]. While clearly remarkable, it is not clear if the reported sensitivity can be reliably maintained in real-life point-of-care applications since, for example, small temperature fluctuations across the Wheatstone bridge can lead to a substantial deterioration of the resistivity measurement precision [25]. The potential of scaling down the sensor size and exploring different sensing modalities leaves ample opportunity for further technology advancement.

**Figure 1:**
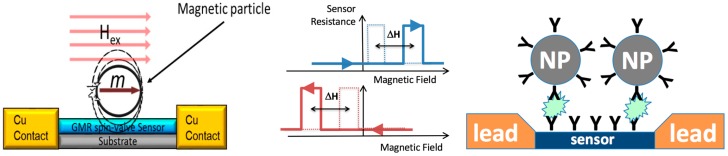
Illustration of magnetic biomolecule sensing using a magnetoresistive sensor. The sensor and magnetic nanoparticles are functionalized with capture antibodies. A target analyte mediates the binding of nanoparticles to the sensor surface. The presence of nanoparticles at the sensor surface is detected via the shift of the switching field (ΔH) under the influence of a magnetic particle.

Magnetic particle detection using a GMR sensor is illustrated in Figure 1. The magnetic stray field generated by a particle or particles captured on or near the sensor surface alters the effective external magnetic field as seen by the sensor. This leads to a shift of the magnetoresistance switching field observed while measuring the dependence of the sensor resistance on the magnetic field. This shift in the sensor switching field enables particle detection. Specific detection can be achieved by functionalizing the sensor and reporter particles with molecular recognition elements (e.g., antibodies as illustrated in Figure 1), chosen to bind to the target to be detected.

This work details design and fabrication of submicron magnetic sensor arrays (400 × 400 nm^2^) capable of detecting individual 200 nm magnetic particles. The ratio of the GMR sensor area in this work to the cross-sectional area of a 200 nm nanoparticle is more than 10 billion fold smaller than the previously-published state of the art. In larger sensors (several microns across), relatively complex measurement and signal processing schemes (e.g., a combination of differential measurement, alternating current (ac) sensing, ac magnetic fields, and direct current biasing fields) is needed to achieve adequate detection levels. Larger sensors also suffer from domain wall noise, limiting the ultimate resolution/sensitivity of the sensor [26]. Furthermore, sensing by large sensors requires a larger number of smaller particles spread across the sensor surface to achieve sufficiently high signal-to-noise ratios (SNRs). In the smaller sensors described in this work, the capture of just one particle gives rise to an easily measurable signal.

## 2. Materials and Methods 

### 2.1. GMR Multilayer Deposition and Characterization 

A GMR spin-valve sensor design was used with a SAF (synthetic antiferromagnet) based bottom reference and free-top magnetic layers with a non-magnetic Cu spacer [27,28]. An AJA-2200 UHV magnetron sputtering system with a base pressure of 1 × 10^−8^ Torr was used to deposit the GMR multilayers onto Si wafers coated with 500 nm of thermally deposited silicon oxide. All depositions were performed under Ar at a 2.5 mTorr working pressure and at room temperature. A magnetic holder was used to apply ~40 Oe magnetic bias field during the deposition of all layers to promote in-plane uniaxial anisotropy in the Permalloy (Ni_80_Fe_20_) layer (see below) [29]. We have verified that this in-plane field has negligible effect on Co layers in the stack. The following GMR stack was used in this work: Ta/Ru/Co/Ru/Co/Cu/Co/Ni_80_Fe_20_/Ta. The deposition rates of Ta, Ni_80_Fe_20_, Co, Ru, and Cu were 0.1 nm/s, 0.06 nm/s, 0.1 nm/s, 0.07 nm/s, and 0.15 nm/s, respectively. The 2.5 nm bottom Ta layer serves as a buffer to promote vertical texturing of the hcp 5 nm Ru seed layer (*c*-axis perpendicular to the plane of the film). The bottom reference magnetic layer in the GMR stack was an asymmetric Co (5 nm)/Ru (0.8 nm)/Co (10 nm) trilayer, where the Ru thickness was optimized to promote antiferromagnetic (AF) coupling between Co layers; AF coupling was verified using trilayers with equal Co layer thicknesses (i.e., Co (5 nm)/Ru (0.8 nm)/Co (5 nm)) [30,31,32]. 

The AF-coupled Co/Ru/Co trilayers exhibited switching field values of ~65 Oe (designed to be higher than the switching field of the top free layer) with the magnetization reversal occurring within a narrow range of applied magnetic fields (<10 Oe). The Cu layer thickness was optimized to maximize the GMR effect in the films before patterning (ΔR/R = 13%). The Co (3 nm) in the top free magnetic layer was used to maximize GMR effect, and a 10 nm Permalloy layer (Ni_80_Fe_20_) with built-in uniaxial anisotropy was added to control the magnetization reversal properties of the resulting Co/Ni_80_Fe_20_ free layer. The Co/Ni_80_Fe_20_ bi-layer was optimized to have a lower switching field (~30 Oe) than the switching field of the bottom reference layer, with the magnetization reversal also taking place within a narrow range of fields (<10 Oe). Magnetization reversal within the narrow field range is essential for the sensing functionality of the developed sensor (see below). The magnetic properties of the as-deposited GMR spin-valve multilayers were characterized using a LakeShore vibrating sample magnetometer (VSM) and four-point probe magnetoresistance measurements.

### 2.2. GMR Sensor Array Fabrication 

**Figure 2:**
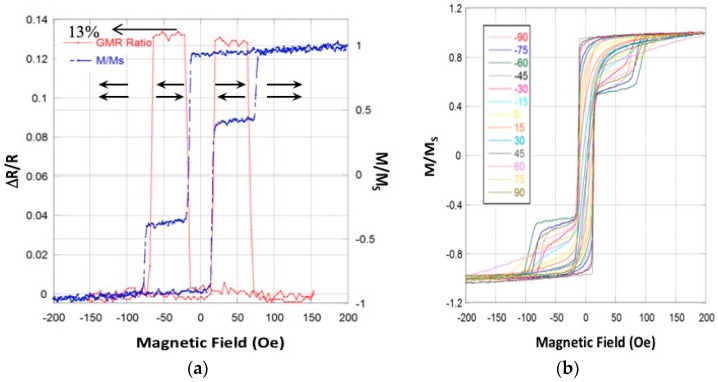
(**a**) The dependence of magnetization (blue dashed line) and giant magnetoresistive (GMR) ratio (red solid line) on applied external magnetic field for the optimized GMR stack used in this work. The low resistance state corresponds to mutually aligned top and bottom magnetic layers; the high resistance state corresponds to top and bottom layers aligned in opposite directions as indicated with the arrows. The GMR stack has a 13% GMR ratio. The parallel and antiparallel alignment is also exhibited in the M-H loops; (**b**) the dependence of magnetization on applied external magnetic field for different field orientations with respect to the easy and hard axes orientations. The 0 and 90 degree notations correspond to the hard and easy axes, respectively.

The normalized magnetization vs. external field (M-H loop) and GMR ratio (ΔR/R) vs. external magnetic field- H loops are shown in Figure 2. Also shown in Figure 2 is the dependence of the GMR multilayer film magnetization on the applied external magnetic field for different field orientations with respect to the easy and hard axis orientations, demonstrating well-defined uniaxial in-plane magnetic anisotropy.

**Figure 3:**
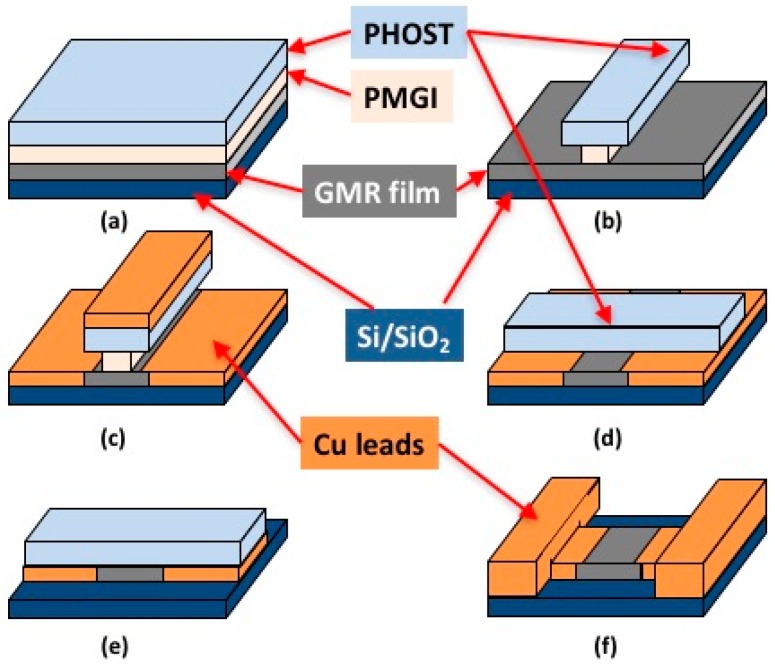
A diagram of the sensor fabrication sequence: (**a**) spin-coat PMGI / PHOST bilayer resist; (**b**) pattern “vertical” lines in PHOST using e-beam lithography, then develop PHOST with PGMEA followed by undercutting PMGI with MF-319; (**c**) transfer PHOST pattern into spin-valve stack (MR) via argon ion milling followed by sputter deposition of Cu leads and PMGI/PHOST lift-off; (**d**) spin-coat PHOST resist, pattern “horizontal” lines using e-beam lithography; develop PHOST with propylene glycol monomethyl ether acetate (PGMEA); (**e**) transfer pattern into spin-valve stack via argon ion-milling; (**f**) strip the resist using oxygen reactive ion etching followed by fabrication of Cu contact pads using photolithography.

The GMR spin-valve stack was patterned using a combination of optical and e-beam lithography (EBL). Self-aligning processes were employed. The fabrication sequence is illustrated in Figure 3 and described below. First, a 150 nm polymethylglutarimide (PMGI) (MicroChem, Westborough, MA, USA) layer [33] was spin-coated onto the GMR film followed by a one minute UV-ozone treatment to reduce surface hydrophobicity and promote the adhesion of a 500 nm polyhydroxystyrene (PHOST) (Sigma-Aldrich, St. Louis, MO, USA) that was spin-coated next. E-beam lithography (JEOL JBX-5500FS) was used to write 1 μm × 400 nm lines into the PHOST resist [34,35] at a dose of 8500 μC/cm^2^, where 400 nm defines the length of the sensor (distance between the current leads) and is aligned with the hard magnetization axis in the multilayer (see above). The PMGI/PHOST bilayer system was then developed in propylene glycol monomethyl ether acetate (PGMEA) (Sigma-Aldrich) for 30 s, followed by a 0.88% tetramethylammonium hydroxide (TMAH) (Shipley MF-319) treatment for 50 s to generate an undercut in the PMGI to facilitate lift-off in the later step of forming Cu leads. Wafers were then ion milled under Ar at 500 V, 34 mA for 80 s to remove the unmasked GMR layers. A 70 nm Cu layer was deposited next by magnetron sputtering followed by lift-off in 3% TMAH.

In the next step, intermediate scale Cu leads were formed. A 500 nm layer of PHOST was spin-coated followed by e-beam writing of 120 μm × 400 nm (for 400 nm wide sensors) or 120 μm × 200 nm (for 200 nm wide sensors) lines perpendicular to and centered at the features defined in the first e-beam lithography step. The PHOST was developed in PGMEA followed by Ar ion milling to remove the unmasked metal layers. The remaining PHOST was then stripped using oxygen plasma etching (Oxford Plasmalab 80 Plus). This processing step forms 400 nm × 400 nm GMR sensors with ~60 μm × 400 nm Cu leads on each side. 

The second larger Cu leads and contact pads were patterned using UV photolithography. PMGI was first spin-coated onto the substrate and baked at 180 °C for 90 s, followed by spin-coating of AZ-1512 (AZ Electronic Materials) photoresist and baking at 90 °C for 90 s. Twelve pairs of contacts were aligned and printed through an ABM 365 nm UV mask aligner, after which samples were post-baked at 120 °C for 90 s. Since TMAH can be used as both AZ-1512 developer and PMGI etchant, the development and etching processes were performed together by immersing the substrate in 3% TMAH solution for 30 s. A 500 nm thick Cu layer was then sputter deposited on the substrate. For the lift-off process, the substrate was immersed in 3% TMAH for 30 s and then in acetone for five minutes with ultrasonic agitation.

**Figure 4:**
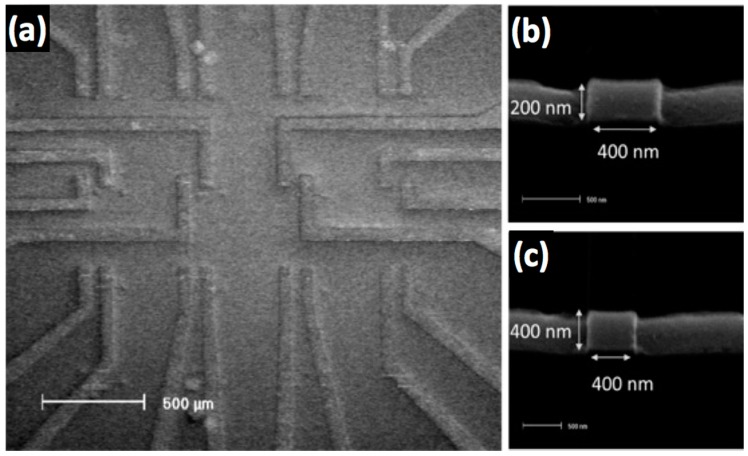
SEM micrograph of (**a**) 12 GMR spin-valve sensors on a chip with Cu contact wires. Closer view of two different dimensions of the sensing area, (**b**) 400 nm × 200 nm and (**c**) 400 nm × 400 nm with their first set of Cu contact wires (the induced easy axis is along the length of these first Cu contact leads).

Using the steps detailed above, 12-sensor arrays of 400 nm-long sensors with two different widths, 200 nm and 400 nm, were built. An SEM micrograph of a GMR spin-valve sensor array of twelve sensors is shown in Figure 4a. Zoomed-in SEM micrographs of a 200 nm × 400 nm sensor and a 400 nm × 400 nm sensor in the array are shown in Figure 4b,c, respectively.

### 2.3. Fe_3_O_4_ Magnetic Nanoparticle Synthesis

A process similar to the one reported by Deng et al. [36] was used to synthesize relatively large Fe_3_O_4_ nanoparticles having diameters of 100 nm and 225 nm. The synthesis involved charging a round-bottomed flask with iron chloride (1.4 g, FeCl_3_·6H_2_O) and 15 mL of ethylene glycol, followed by the addition of sodium acetate (3.6 g) to obtain a brown solution. The solution was stirred for an additional 30 min and then injected at once into a round-bottomed flask containing a vigorously stirred solution of PVP (0.40 g) in 35 mL of ethylene glycol heated to 180 °C. This mixture was then stirred at 180 °C for 6 or 24 h to obtain 100 nm and 225 nm particles, respectively, during which a black precipitate was obtained. The black precipitate was washed multiple times with ethanol and Milli-Q water and dried under vacuum at room temperature. 

## 3. Results

### 3.1. GMR Sensor Array Characterization

**Figure 5:**
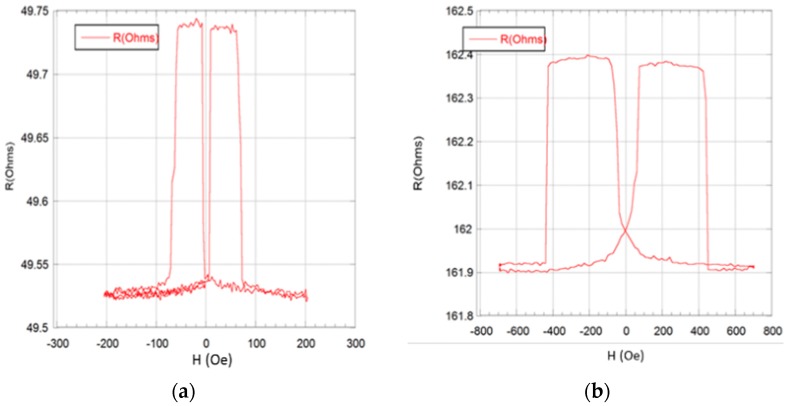
Dependence of sensor resistance on magnetic field for the 400 nm × 400 nm (**a**) and the 200 nm × 400 nm (**b**) sensors.

A 0.2 mA DC current was used for the resistance measurement while applying an external magnetic field. The applied field utilized was a triangle wave swept from +800 Oe to −800 Oe and back to +800 Oe at a frequency of 0.05 Hz. It took 30 s to probe a single sensor. Typical sensor responses to an external magnetic field applied along the length of the sensor (ΔR-H loops) are shown in Figure 5. The switching from antiparallel to parallel magnetization orientations of the top and bottom layers (switching from high to low resistance state) takes place at ~65 Oe for both the continuous film. While the switching field for the 400 nm × 400 nm sensors was also in ~65 Oe range, due to patterning process imperfections, this value could be as high as 120 Oe. However the same switching takes place at ~420 Oe for the 400 nm × 200 nm sensor. The significant change in the switching field is due to shape anisotropy (demag) in the latter sensors. Furthermore, the switching from low to high resistance state for both the continuous film and for the 400 nm × 400 nm sensors occurs within a fairly narrow range of applied fields, while the same switching for 400 nm × 200 nm sensors is spread over a ~125 Oe field range due to the demagnetizing field. The observed GMR ratio in the patterned sensors is ~0.5% and ~0.3% for the 400 nm × 400 nm and the 400 nm × 200 nm sensors, respectively. The smaller GMR ratio in the patterned sensors than in the continuous films is due to electrical lead and contact resistance. Although the magnetoresistive response (ΔR/R) of the sensors was relatively small, the step-like change in the resistance used for particle detection was easily resolvable without any signal processing. The sensor response for many consecutive measurements showed that the switching field reliably fell within ±5 Oe. The resolution of the measurement system used in this work is more than sufficient to detect magnetization switching reliably. 

A 400 nm × 400 nm sensor array was chosen as the platform for nanoparticle detection due to its well-defined high-to-low and low-to-high resistance state switching as well as its low switching fields. The shifts in both high-to-low and low-to-high resistance switching fields can be used to detect nanoparticles. Also, the lower switching field reduces the design and power requirements for the external magnetic field source.

### 3.2. Fe_3_O_4_ Magnetic Nanoparticle Characterization

**Figure 6:**
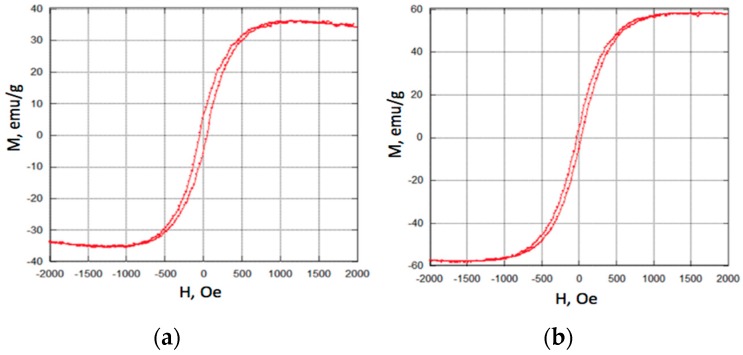
Magnetization vs. magnetic field (M-H loops) for 100 nm (**a**) and 225 nm (**b**) Fe_3_O_4_ nanoparticles.

The spinel crystal structure of the synthesized Fe_3_O_4_ nanoparticles was confirmed using X-ray diffraction (XRD), and the particle size was measured using scanning electron microscopy. The M-H loops of the both sets of particles are shown in Figure 6. Consistent with previously published data, the larger particles exhibited higher saturation magnetization, likely due to better developed crystallinity [37,38,39]. Both 100 nm and 225 nm particles saturated at approximately 1000 Oe applied field.

### 3.3. Application to Nanoparticle Detection

**Figure 7:**
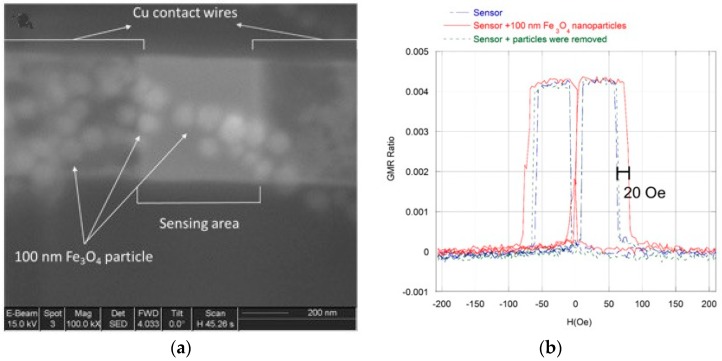
(**a**) SEM micrograph of several 100 nm Fe_3_O_4_ particles deposited on the 400 nm × 400 nm sensing area; (**b**) GMR profile change of a 400 nm × 400 nm sensor depending on whether several 100 nm Fe_3_O_4_ nanoparticles were deposited on the sensing area. The switching field of the sensor covered with the particles increased by ~20 Oe. As the particles were removed, the GMR profile returned to its initial state.

A 100-μL volume of a 0.18 mg/mL suspension of 100 nm Fe_3_O_4_ nanoparticles in ethanol was pipetted onto the sensor array, and the ethanol was allowed to evaporate to deposit the particles onto the surface. Since the location of the deposited particles on the sensor array surface was random, the procedure had to be repeated multiple times until the particles landed on the GMR sensor surface. An SEM image of ~10 nanoparticles over a 400 nm × 400 nm sensor is shown in Figure 7. Aggregation of nanoparticles was observed in the majority of our experiments. The aggregation likely takes place during the drying process because the nanoparticles (1) are mutually attracted due to remnant magnetization and (2) are attracted to the magnetic sensors due to stray fields generated by sensors. The combination of the two effects improve the probability of the particles landing on the sensors. 

The sensor response was evaluated before particle application, with the particles present, and after the particles were washed away with ethanol. As shown in Figure 7, the GMR sensor high-to-low resistance switching field increased by ~20 Oe in the presence of the particles. It is important to point out that the presence of magnetic particles over adjacent leads can affect the readout signal. However, this contribution is minor as the magnetic field strength generated by these particles decays rapidly (as a power of 3) with the distance.

**Figure 8:**
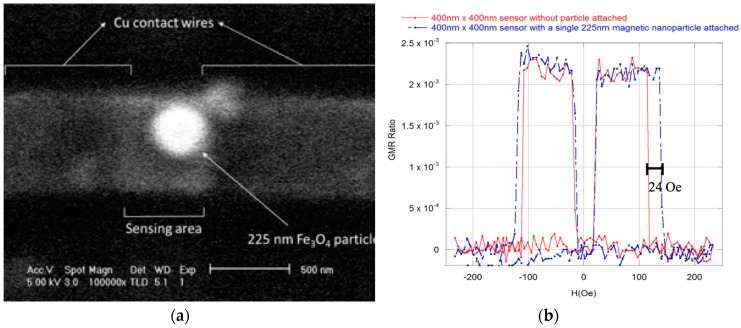
(**a**) SEM micrograph of a single 225 nm Fe_3_O_4_ nanoparticle deposited on the 400 nm × 400 nm sensing area. (**b**) GMR profile before and after a single 225 nm Fe_3_O_4_ particle deposition on a 400 nm × 400 nm sensor. A shift of ~24 Oe in the positive value of the switching field and a ~10 Oe change in the negative value of the switching field was observed. The asymmetric change is due to asymmetric positioning of the particles over the sensor surface.

Next, we evaluated the feasibility of *single-nanoparticle* detection. An approach similar to the one above was employed using 225 nm Fe_3_O_4_ particles (100 nm particles did not generate sufficient stray field to give a reliably measurable shift in the GMR sensor switching field). The response of the sensor to the application of a single 225 nm nanoparticle is shown in Figure 8. A 24 Oe shift in the sensor switching is observed at the positive values of the field. This shift is smaller for the negative values of the field due to asymmetric positioning of the particle over the sensor surface. Significantly, the detection of a single nanoparticle is a TRUE/FALSE type of event where it is the presence of the nanoparticle that is being detected rather than, for example, the absolute value of the field switching field shift. As long as the particle is located over the sensor or in close proximity to the sensor, the shift in the switching field will take place manifesting particle detection. This feature makes the developed sensor a pseudo-digital rather than an analog detection device.

## 4. Discussion and Conclusions

A sub-micron GMR spin-valve array was prepared and used to demonstrate the detection of individual 225 nm Fe_3_O_4_ nanoparticles as well as small numbers of 100 nm Fe_3_O_4_ nanoparticles. This sensing scheme provides a pseudo-digital signal reporting the presence of a single nanoparticle. As seen from the data, the exact particle position over the sensor surface affects the details/profile of the readout signal. However, since the sensor is designed to report the presence or absence of the particles, any detectable shift in the switching field of individual sensors manifests a positive detection event. These sensors are not designed to, for example, count the number of particles in a sample. To achieve further increases in nanoparticle sensitivity using the specific detection approached utilized in this work, namely the detection of the shift in the switching field, the sensor dimensions will need to be reduced further. For example, we expect that a sensor array with 200 nm × 200 nm sensors will be more than adequate to detect individual 100 nm nanoparticles.

The sensors were built as a proof-of-concept of single nanoparticle detection using simple dc current readout electronics. They are inexpensive to manufacture and can be arrayed to increase the effective detection area and/or multiplex capabilities. While corrosion protection layers were not used in this work, ultra-thin alumina coatings (<50 nm) have been demonstrated to provide adequate protection in electrolytic aqueous solutions, such as phosphate-buffered saline (PBS), commonly used for biosensing environments [40]. It should be noted that the thickness of corrosion protection layers should be minimized to minimize the spacing between the nanoparticles and sensors. Significantly, the reader electronics (current source, Helmholtz coil, and voltmeter) can be inexpensively packaged into a portable or mobile system for point-of-care diagnostics or in-field analysis.
